# Late Central Airway Toxicity after High-Dose Radiotherapy: Clinical Outcomes and a Proposed Bronchoscopic Classification

**DOI:** 10.3390/cancers13061313

**Published:** 2021-03-15

**Authors:** Juliët E. van Hoorn, Max Dahele, Johannes M. A. Daniels

**Affiliations:** 1 Department of Pulmonary Medicine, Cancer Center Amsterdam, Amsterdam University Medical Center, De Boelelaan 1117, 1081 HV Amsterdam, The Netherlands; j.daniels@amsterdamumc.nl; 2 Department of Radiation Oncology, Cancer Center Amsterdam, Amsterdam University Medical Center, De Boelelaan 1117, 1081 HV Amsterdam, The Netherlands; m.dahele@amsterdamumc.nl

**Keywords:** radiotherapy, bronchoscopy, airway toxicity, radiation damage, bronchoscopic classification, clinical outcomes, complication

## Abstract

**Simple Summary:**

High-dose radiotherapy is frequently used to treat lung cancer, however, it can cause serious central airway toxicity. Although radiation toxicity of the lung parenchyma has been studied extensively, relatively little has been published on bronchoscopic findings in the central airways and no standard classification/reporting system exists. With the growing use of high-dose (chemo)radiotherapy and high-dose hypo-fractionated radiotherapy in close proximity to central airways, as well as potential interactions with new systemic therapies, the risks and incidence of central airway toxicity may increase. In this retrospective study, we analyzed patient characteristics and clinical outcomes of 70 patients with central airway toxicity after high-dose radiotherapy. Furthermore, we analyzed the post-radiotherapy bronchoscopic images to identify main patterns of airway toxicity. We identified luminal stenosis and vascular changes as the two main patterns and have proposed a classification system. Preliminary analysis suggests that the pattern and severity of radiation toxicity may be of prognostic value.

**Abstract:**

The study’s purpose was to identify the bronchoscopic patterns of central airway toxicity following high-dose radiotherapy or chemoradiotherapy, and to look at the consequences of these findings. Our institutional bronchoscopy database was accessed to identify main patterns of airway toxicity observed in a seven-year period. A total of 70 patients were identified with central airway toxicity, and the findings of bronchoscopy were used to derive a classification system. Patient characteristics, time from radiotherapy to toxicity, follow-up and survival were retrospectively analyzed. *Results:* The main bronchoscopic patterns of airway toxicity were vascular changes (telangiectasia, loss of vascularity, necrosis) and stenosis of the lumen (moderate, severe). Indications for bronchoscopy were airway symptoms (*n* = 28), assessment post-CRT/surgery (*n* = 12), (suspected) recurrence (*n* = 21) or assessment of radiological findings (*n* = 9). Stenosis was revealed by bronchoscopy at a median time of 10.0 months (IQR: 4–23.5) after radiotherapy and subsequent follow-up after identification was 23 months (IQR: 1.5–55). The corresponding findings for vascular changes were 29 months (IQR: 10.5–48.5), and follow-up after identification was nine months (IQR: 2.5–19.5). There was a statistically significant difference in survival rates between patients with necrosis and telangiectasia (*p* = 0.002) and loss of vascularity (*p* = 0.001). Eight out of 10 deceased patients with telangiectasia died of other causes and 4/8 patients with necrosis died of other causes. We identified two main patterns of central airway toxicity visualized with bronchoscopy after high-dose radiotherapy or chemoradiotherapy, and propose a bronchoscopic classification system based on these findings. Preliminary analysis suggests that the pattern and severity of radiation damage might be of prognostic value. Prospective data are required to confirm our findings.

## 1. Introduction

High-dose radical radiotherapy (RT), including conventionally fractionated (e.g., 2Gy/fraction) RT with or without chemotherapy and stereotactic body RT (SBRT), is a guideline recommended treatment for early-stage and locally advanced lung cancer, as well as for metastases in the lung and mediastinal lymph nodes [1,2,3,4]. When applied to tumors in close proximity to the central airways (not only lung tumors but also, e.g., mediastinal and esophageal tumors) [5,6,7], high-dose RT can cause clinical toxicity. Previous studies have reported sequelae ranging from mild airway narrowing [8] to fistula and necrosis and, in some cases, fatal events such as massive hemoptysis [5,9,10,11,12]. With the growing use of SBRT for central lung tumors [3,13,14,15], an increasing number of new targeted lung cancer treatments with unknown effects on irradiated tissues/interactions with radiation [15,16,17,18,19,20], and an improvement in prognosis (e.g., for patients with metastatic disease and certain driver mutations, and following the introduction of adjuvant immunotherapy for locally-advanced disease), the incidence of these complications may rise in the future. Although radiation toxicity of the lung parenchyma has been studied extensively, relatively little has been published on bronchoscopic findings in the central airways and no standard classification/reporting system exists. The purpose of this retrospective study is (1) to bronchoscopically identify specific patterns of toxicity to the central airways after high-dose (chemo)RT that can form the basis for a classification system, and (2) to explore the natural history and consequences of these bronchoscopic findings.

## 2. Materials and Methods

This study was conducted with the approval of the institutional medical ethics committee.

### 2.1. Data Collecting and Classification System

The Amsterdam UMC is a tertiary referral center in North Holland and an institutional bronchoscopy database has been maintained since 2012. Following ethics approval, we queried this database for a 7-year period to identify patients with findings of central airway toxicity following any form of high-dose thoracic radiotherapy and a bronchoscopic examination. Bronchoscopic reports that mentioned any potential radiation induced toxicity and the accompanying bronchoscopic images were used to identify patients for the analysis. The location of the treated area was categorized as trachea, central and peripheral. We defined a central tumor as a tumor located within the region of the central airways: from the trachea up to and including the segmental bronchi. The descriptions of the changes in the bronchial tree were recorded and the images were inspected to identify different patterns of radiation damage that could be used to form the basis for a classification system and to grade their extent. Stenosis was assessed by one observer using bronchoscopic videos, photos and reports of the bronchoscopy. If there was any doubt, images were reviewed with a second observer.

Using the bronchoscopy database, institutional electronic patient record system and institutional radiotherapy information system the patient characteristics, treatment details, indications for bronchoscopy and follow-up data, were recorded.

### 2.2. Follow-Up

Patients received standardized follow-up according to national guidelines specific to the cancer type and stage. Bronchoscopy was performed when clinically indicated (e.g., symptoms or suspicion of cancer recurrence) or if imaging studies were suspicious for radiation damage.

### 2.3. Statistical Analysis

Time to airway toxicity was measured from the start date of radiotherapy to the date of bronchoscopy that showed toxicity. Follow-up time was defined as the time from the date of bronchoscopy to the last date of contact or the date of death. The Kaplan–Meier method was used to measure survival time after bronchoscopic diagnosis of toxicity, and the log-rank test (pairwise over strata) was used to compare survival by the degree of airway toxicity (extracted from the classification system). Patients who had tumor recurrence, proven by biopsy or high suspicion on imaging, were not included in the survival analysis. For the survival analysis, 2nd and 3rd degree vascular changes were combined since patient groups were small. IBM SPSS Statistics for Mac, Version 24.0, was used for all the statistical analyses.

## 3. Results

### 3.1. Patient and Treatment Characteristics

A total of 70 patients, three of whom we have previously described in a case series [10] and who were treated between 2008 and 2018, met the study inclusion criteria. Patient, tumor and treatment characteristics are described in Table 1. All patients were treated with high-dose RT (referring here to a dose of/biologically equivalent to ≥50 Gy in 2Gy/fraction). Of the 70 patients, 10 had undergone treatment with SBRT, 14 with conventional/moderately hypo-fractionated RT alone and 46 with (concurrent or sequential) chemoradiotherapy (CRT). The most common treatment schedules used were 33 × 2 Gy for conventionally fractionated treatment and 12 × 5 Gy for hypofractionated treatment. For 22 patients irradiated elsewhere the exact start date of RT was missing; for these patients the middle of the month or year of RT treatment was used in case only the month or year was known. Indications for bronchoscopic evaluation after RT are shown in Table 1. A total of 23 patients with evidence of tumor recurrence, proven by biopsy or with highly suspicious imaging at time of bronchoscopic evaluation, were excluded from the survival analyses which were based on 47 patients. Chronic obstructive pulmonary disease (COPD) was the most common comorbidity (*n* = 27). At the time of data analysis, 36 patients were deceased.

### 3.2. Bronchoscopic Findings and Proposed Classification System

**Figure 1:**
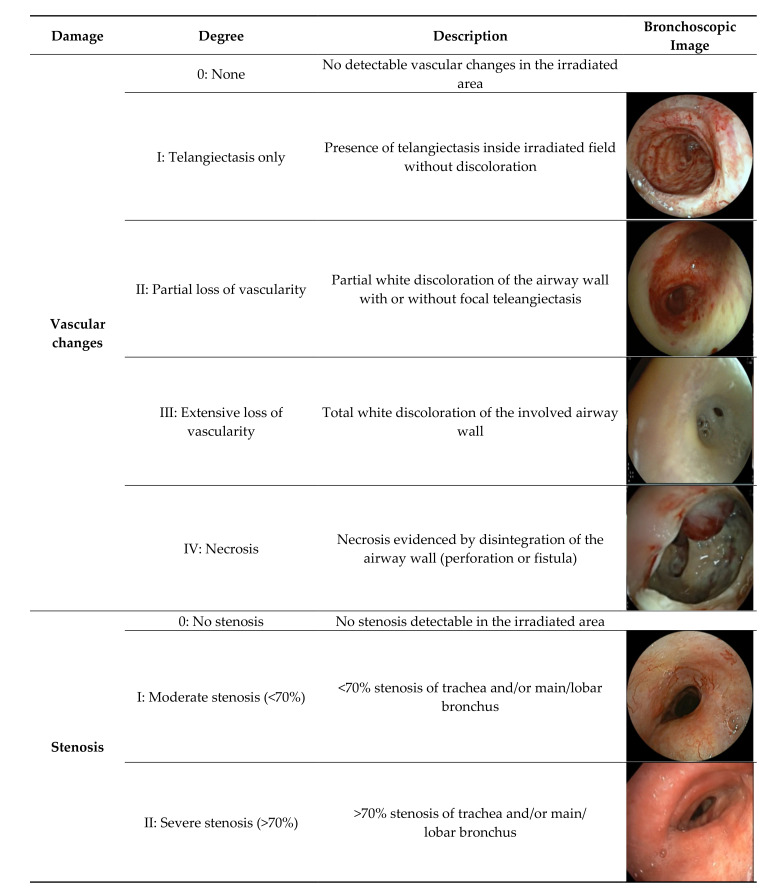
Proposed classification system based on bronchoscopic findings from 70 patients.

The most common patterns of toxicity observed during bronchoscopy (*N* = 70) were (1) vascular changes and (2) luminal stenosis. Based on the institutional experience, a toxicity classification system based on these two patterns was proposed (Figure 1). Findings of vascular changes were further separated into telangiectasia, partial and extensive loss of vascularity, and necrosis within the irradiated field. Table 2 shows the frequency of each degree of toxicity for the different types of radiation treatment.

Partial or extensive loss of vascularity is evidenced by respectively partial or total white discoloration of the airway wall. White mucus or pus on the airway wall can be mistaken for discoloration. Consequently, we assessed if the white area could be mobilized/flushed away before defining it as airway wall discoloration. Necrosis is evidenced by disintegration of the airway wall (perforation or fistula). Stenosis was defined as none, moderate or severe, with the estimated cut-off value between moderate and severe stenosis being a 70% reduction in luminal diameter, based on the Meyer–Cotton classification for subglottic stenosis [21].

### 3.3. Vascular Changes

Twenty-three out of 70 patients (32.9%) developed telangiectasia, 18 had partial loss of vascularity, 11 extensive loss and 11 necrosis of the airway wall. Vascular changes without any degree of stenosis were observed in 25 patients. Of the latter, five underwent bronchoscopy for hemoptysis. The indications for bronchoscopy for each pattern of toxicity specifically are shown in Table 3.

Table 4 shows the intervals from radiotherapy until toxicity and from toxicity until last contact or death (*N* = 70). In patients with any degree of vascular changes, without stenosis, toxicity was identified at bronchoscopy a median of 29.0 months (IQR: 10.5–48.5) after RT and follow-up of the vascular changes was a median of nine months (IQR: 2.5–19.5).

**Figure 2:**
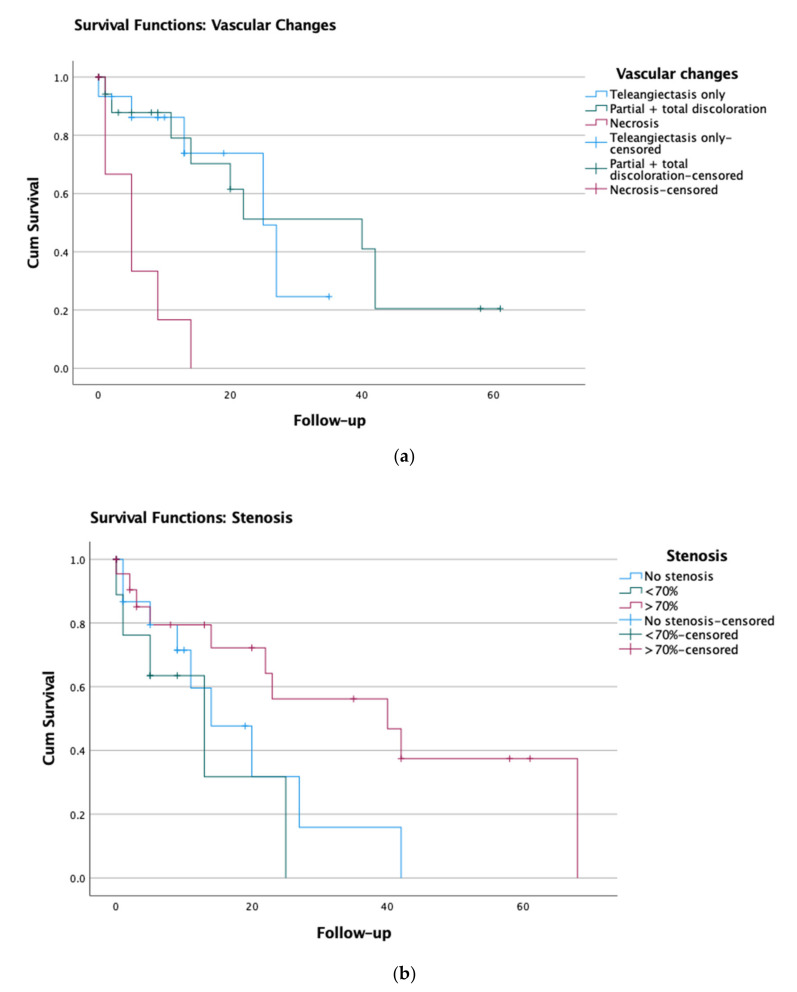
Kaplan–Meier survival analysis for (**a**) vascular changes and (**b**) airway stenosis (based on *n* = 47).

Study of patients exhibiting the different degrees of vascular changes revealed a statistically significant difference in survival between those with degree I (telangiectasia only) and IV (necrosis; *p* = 0.002), and between patients exhibiting degrees II (partial loss of vascularity)/III (extensive loss of vascularity) and IV (*p* = 0.001). No statistically significant difference in survival was found between patients with degrees I and II/III (*p* = 0.661) (Figure 2a). At 12 months after bronchoscopy, 13.3% of patients with telangiectasia, 15.7% of patients with partial and total loss of vascularity and 62.5% of patients with necrosis (*N* = 8) were deceased.

At the time of data analysis, a total of 32 patients with vascular toxicity were deceased. Of the 10 patients with grade I vascular changes, 8/10 died of other causes (such as progression of disease or comorbidities) and in two the cause of death was unknown. Three out of six patients with grade II vascular changes died of other causes and in three the exact cause of death was unknown. Four out of eight deceased patients with grade III vascular changes died of these changes (e.g., fatal hemoptysis) and in four the exact cause of death was unknown. Of the patients with grade IV vascular changes, four died of these changes and four died of other causes.

### 3.4. Bronchial Stenosis

Stenosis was found in 45/70 patients (64.3%) with airway toxicity, moderate in 13 and severe in 32. Severe stenosis was found in nine patients who underwent bronchoscopy after complaints of dyspnea and in two patients who presented with respiratory failure (Table 3.) The location of airway stenosis was tracheal in eight patients, central in 30 (main and lobar bronchus) and at the level of the segmental bronchus in four patients.

In most patients the site where airway stenosis developed was either at the location of the original tumor (*N* = 18) or nearby (*N* = 20; e.g., radiation of a distal tracheal tumor leading to stenosis of the right main bronchus). In some patients, stenosis developed at a site not directly related to the original tumor location (*N* = 6; e.g., stenosis of the left main bronchus after RT for an esophagus tumor) and in one patient the original tumor site was unknown.

Bronchial stenosis was revealed by bronchoscopy at a median time of 10.0 months (IQR: 4–23.5) after RT and follow-up after diagnosis of the stenosis was 23 months (IQR: 1.5–55; Table 4). Appendix A provides more information about the outcome of patients who developed stenosis only.

There was no statistically significant difference in survival between patients with the different degrees of stenosis: between degree 0 (none) and I (moderate; *p* = 0.328); degree 0 and II (severe; *p* = 0.078); degree I and II (*p* = 0.063) (Figure 2b). At time of data analysis, four patients who developed stenosis without vascular changes were deceased. Of these patients, one died of the bronchial stenosis and three patients died of other causes.

### 3.5. Combined Stenosis and Vascular Changes

Patients with both vascular changes and moderate or severe stenosis were identified at a median time of 28 months (IQR: 14.5–70.0) and 22.5 months (IQR: 13.0–60.0) after RT, respectively. For patients exhibiting these changes, follow-up after bronchoscopic diagnosis was five months (IQR: 0–11.0) and 11 months (IQR: 2.0–23.0), respectively (Table 4).

## 4. Discussion

This study shows that bronchoscopic findings of airway toxicity after radiotherapy fall into two main categories: stenosis and vascular changes. We propose a classification system based on these features and their severity. Initial evaluation suggests that this classification system has prognostic value: survival for patients with necrosis and airway perforation/fistulation was significantly poorer than for patients with stenosis or milder degrees of vascular changes (telangiectasia/loss of vascularity) and most patients with telangiectasia died of other causes, whereas half of the patients with necrosis died from it. In addition, the timeline of various changes is seen to vary, with telangiectasia being found relatively late after RT, and necrosis earlier (Table 4). One explanation for this could be that severe patterns of toxicity, e.g., necrosis with airway perforation or fistula, are symptomatic whereas mild vascular changes are not and therefore probably revealed at a later point in time at which bronchoscopy is performed for another indication than airway symptoms.

Although the degree of stenosis does not seem to be of prognostic value for survival, (severe) stenosis may cause troubling clinical symptoms and lead to secondary complications such as obstructive pneumonia and atelectasis requiring intervention (e.g., dilatation and/or endobronchial stent placement), adversely affecting functional status and quality of life. Stenosis at the segmental level is presumably less relevant for most patients. In this study, four patients with stenosis at the segmental level were included, however vascular toxicity was the primary reason for inclusion and segmental stenosis was an additional finding (e.g., in one patient who presented with hemoptysis bronchoscopy revealed telangiectasia, considered to be the cause of hemoptysis, and also showed a segmental-level stenosis).

Further work is needed to identify relevant etiological factors and to better understand the pathogenesis of airway toxicity. The classification system may be useful to enhance clarity when describing airway toxicity, and can be used to keep track of longitudinal changes in individual patients. There is a suggestion in Table 2 that severe (grade 2) stenosis may be more common after SBRT and that serious (grade III/IV) vascular changes may be more common after RT and CRT. The very high biological doses used in SBRT could be a rational explanation if indeed there is an excess of stenosis; and RT and CRT are expected to be used in more central locations than SBRT—these more central airways might be more prone to vascular damage. These hypotheses merit further investigation.

The study has a number of limitations. It is retrospective and there is referral bias. Not all irradiated patients undergo bronchoscopy, while symptomatic patients and patients with a possible recurrence are more likely to undergo bronchoscopy. Therefore, even though patients with a recurrence were excluded from the survival analyses, generalizability to the whole population of irradiated patients is limited because the incidence and prognosis may not accurately reflect findings in an unselected population of asymptomatic patients. In addition, the limited sample size and inclusion of patients with different types of treatment (RT, SBRT and chemo-RT), and types and stages of disease, precludes multivariate data analyses and limits our ability to reliably identify risk factors for toxicity and predictors of outcome. Loss of patients from follow-up may have led to an under-reporting or less severe scoring of toxicity. Since this is a retrospective study, lung function and exercise tests to assess the functional impact of the bronchoscopic findings were unfortunately not routinely available. The impact of various degrees of toxicity on lung function, quality of life and exercise capacity merits investigation.

However, these limitations should be seen in context and placed in perspective. The purpose of this study was not to define the true incidence of airway toxicity or to accurately determine survival. This is an exploratory and hypothesis generating data analysis with the purpose of identifying and classifying patterns of airway toxicity after RT as a basis for further prospective research.

Furthermore, we believe that the study provides useful information given the scarcity of existing studies that look at bronchoscopic findings of airway toxicity and its natural history and consequences. To the best of our knowledge, this is the first bronchoscopic classification system specifically proposed for airway toxicity after RT.

There is a need to validate these findings and further investigate risk factors (e.g., chemotherapy, targeted therapy, smoking, diabetes etc.), with a sufficiently large prospective cohort of unselected patients. Furthermore, studies relating bronchoscopic findings to clinical symptoms and quality of life are needed, and the pathogenesis of toxicity needs to be understood. This would help with the development of preventative and therapeutic strategies.

### 4.1. Mechanisms of Toxicity

Although radiation is considered as the primary etiologic factor, the combination of RT with chemotherapy and/or new systemic therapies may increase the risk and incidence of serious airway toxicity [16,17,18,22]. The extent by which this risk increases is unknown. The exact etiologic mechanisms of airway toxicity are also yet to be elucidated. Analogous with radiation dermatitis, connective tissue atrophy, fibrosis, sclerosis, vascular damage and neovascularization may all play a role [23].

### 4.2. Interventions to Reduce Airway Toxicity

There are limited options for preventing and managing RT-related airway toxicity. Radiation dose reduction and conventionally fractionated treatment schedules may help to reduce the risk of toxicity, however, they may also result in increased local failure and poorer cancer control. Virtual bronchoscopy-guided treatment planning which aims to minimize the dose to individual airway segments, and therefore potentially reduce the risk of airway toxicity, has been described [24,25]. However, the impact on toxicity needs to be defined.

Regarding post-treatment management, bronchoscopic intervention with balloon dilatation and stent placement is an effective and established method to relieve symptoms of airway stenosis but can cause irritation, mucus retention and infections [5]. There are also surgical interventions for airway toxicity. Dickhoff et al. have described the surgical treatment of complications (e.g., stenosis, hemorrhage, fistulas) following high-dose CRT. Surgery is often the only remaining treatment for patients with severe, irreversible toxicity, although most patients may ultimately be deemed inoperable [26]. CRT before lobectomy may adversely affect the bronchial mucosal blood flow, and therefore impair healing of the bronchial stump. Tissue reinforcement/buttressing of the irradiated bronchus after lobectomy is therefore recommended and practiced by some surgeons [22,27,28]. Appendix B provides an overview of post-treatment interventions for the patients in this study.

Immunosuppressive medication could be of use if inflammation plays a role in the development of airway toxicity. However, this may also increase the risk of infection if tissue is poorly vascularized. In addition, a side effect of immunosuppressive therapy could be subversion of immune surveillance, potentially increasing the risk of cancer recurrence. Mesenchymal stem cells for radiation-induced toxicity have not been studied yet in the airways but have shown some promise in a recent trial for radiation-induced xerostomia after head and neck radiotherapy [29,30]. This strategy merits further consideration.

## 5. Conclusions

Analysis of post-radiotherapy bronchoscopic images identified two main patterns of airway toxicity, luminal stenosis and vascular changes, and we have proposed a classification system. In addition, preliminary analysis suggests that the pattern and severity of radiation toxicity may be prognostic.

## Figures and Tables

**Table 1:** Patient, tumor and treatment characteristics (*N* = 70). NSCLC: non-small cell lung cancer; SBRT: stereotactic body radiotherapy; RT: conventional/moderately hypo-fractionated radiotherapy alone; CRT: concurrent or sequential chemoradiotherapy.

Characteristics	No.
Gender	
Male	42
Female	28
Age (years)	
Median (range)	64 (42–80)
Location of malignancy	
Trachea	8
Central (up to and segmental bronchi)	21
Peripheral	36
Esophagus	3
Mediastinal lymph node	1
Pathology	
NSCLC	64
Metastases	2
Other	4
Type of RT	
SBRT	10
Conventional	14
Chemoradiotherapy (CRT)	46
Indication for bronchoscopy	
Airway symptoms	28
Assessment post CRT/surgery	12
Suspected tumor recurrence	21
Radiological findings	9
Recurrence at time of bronchoscopy	
Free of recurrence	47
High suspicion of recurrence on imaging	9
Proven recurrence with biopsy	14

**Table 2:** Frequency table for degree of toxicity per type of radiation treatment. SBRT: stereotactic body radiotherapy; RT: conventional/moderately hypo-fractionated radiotherapy alone; CRT: concurrent or sequential chemoradiotherapy.

Damage	Degree	SBRT	RT	CRT
**Vascular changes**	0: None	0	2	3
	I: Telangiectasis only	5	3	17
	II: Partial loss of vascularity	3	3	12
	III: Extensive loss of vascularity	1	4	6
	IV: Necrosis	1	2	8
**Stenosis**	0: No stenosis	3	5	17
	I: Moderate stenosis (<70%)	1	1	11
	II: Severe stenosis (>70%)	6	8	18
**Total**		10	14	46

**Table 3:** Indications for bronchoscopy (*N* = 70).

Indication for Bronchoscopy	Vascular Changes Only *N* = 25	<70% Stenosis *N* = 13	>70% Stenosis *N* = 32
Hemoptysis	5	3	3
Dyspnea	1	1	9
Respiratory insufficiency	1	1	2
Recurrent infections	0	0	2
Assessment post-CRT or surgery	3	2	7
Assessment (suspected) recurrence	11	1	3
Intervention recurrence (diathermy/debulking)	2	2	2
Assessment of radiological findings suspicious for RT damage	2	3	4

**Table 4:** Median intervals (months (IQR)) between radiotherapy and toxicity; toxicity and last contact/death (*n* = 70 patients). RT: radiotherapy.

Damage	Degree	RT-Toxicity	Follow-Up
**Vascular changes**	0: None (*N* = 5)	10.0 (4.0–23.5)	23.0 (1.5–55.0)
	I: Telangiectasis only (*N* = 14)	47.0 (26.0–88.0)	10.0 (5.0–16.0)
	II: Partial loss of vascularity (*N* = 10)	23.0 (12.5–84.5)	13.5 (4.0–31.0)
	III: Extensive loss of vascularity (*N* = 9)	19.0 (12.0–37.0)	1.0 (0–11.0)
	IV: Necrosis (*N* = 10)	13.0 (6.0–23.0)	5.0 (1.0–10.0)
**Stenosis**	0: No stenosis (*N* = 19)	29.0 (10.5–48.5)	9.0 (2.5–19.5)
	I: Moderate stenosis (<70%) (*N* = 8)	28.0 (14.5–70.0)	5.0 (0–11.0)
	II: Severe stenosis (>70%) (*N* = 21)	22.5 (13.0–60.0)	11.0 (2.0–23.0)

## Data Availability

The data are not publicly available due to privacy restrictions.

## Appendix A. Outcome for Patients with Stenosis Only

**Stenosis Degree**	**Indication Bronchoscopy/Symptoms**	**Intervention**	**Follow-Up after Bronchoscopy**	**Status**
>70%	Post-treatment inspection	-	42 months till last contact.	Alive
>70%	Impending respiratory insufficiency	Dilatation and stent placement 5 months post-RT	Died 68 months after bronchoscopy. Probable cause of death related to heart problems, not airway stenosis.	Dead
>70%	Severe tracheomalacia and stenosis, respiratory insufficiency	Tracheostomy, canula	Died 23 months after bronchoscopy. Repeated IC admissions for respiratory insufficiency. Palliative care for progressive dyspnea.	Dead
>70%	Severe dyspnea	Stent placement 2 years after CRT and immuno-therapy	Died 9 days after bronchoscopy. Palliative care for progressive dyspnea.	Dead
>70%	Post-treatment inspection. Dyspnea in combination with COPD exac-erbation.	Antibiotics	Died 3 months after bronchoscopy	Dead

## Appendix B. Post-Radiation Interventions for Airway Toxicity

No intervention No intervention necessary Deemed inoperable No bronchoscopic options left (e.g., recurrent stenosis)	51 31 12 8
Dilatation	2
Dilatation and stent placement	7
Stent placement	4
Pneumonectomy/lobectomy	2
Reinforcement surgery	2
Tracheostoma/canula	2
